# The Critical Role of Permanent Voucher Specimens of Hosts and Vectors in Public Health and Epidemiology

**DOI:** 10.3201/eid1602.091241

**Published:** 2010-02

**Authors:** A. Townsend Peterson

**Affiliations:** University of Kansas, Lawrence, Kansas, USA

**Keywords:** Voucher specimens, host, vector, museum, public health, epidemiology, biodiversity, commentary

Zoonotic disease transmission systems constitute sets of interacting species, ranging from pathogens in wildlife reservoirs and transmitted directly to humans (*1*), pathogens in wildlife reservoirs and transmitted to humans by vectors (*2*), to pathogens in complex systems of multiple interacting definitive hosts, intermediate hosts, and vectors (*3*). Because these systems are so tightly linked to biodiversity, studies must include sampling of diverse species. Emerging Infectious Diseases journal is replete with such studies documenting the circumstances surrounding a disease case or outbreak. However, in developing these studies, researchers have been remiss regarding 1 major element of documentation of their work. Other elements are documented carefully. For example, when sequence data are extracted, primer sequences are presented in the Materials and Methods section, and numbers are given of sequences deposited in the GenBank database. Biodiversity samples, in contrast, are often identified cursorily or incompletely, and the documentation (i.e., the host or vector animal from which the pathogens were isolated) is discarded. I suggest to the public health, epidemiology, and disease ecology communities that careful biodiversity documentation is critical to full description of disease research. As a routine part of the research process, voucher specimens should be deposited in an appropriate scientific collection, and catalog numbers reported in publications,

Disease studies that lack careful biodiversity documentation are numerous, even in the recent literature. In the August 2009 issue of this journal, I found at least 4 articles that report sampling of hosts or vectors, yet make no mention of vouchers (*4**–**7*). Quite simply, and with few counterexamples (*8*), host and vector information is ignored, as if all identifications are perfect and complete and as if nothing remains to be learned from further study of the samples.

The reality, however, is quite different. First, technologies for diagnosis and testing have evolved considerably and will continue to evolve, with each iteration providing more complete information and insight into the pathogens present. The failure to preserve voucher specimens, however, makes such retesting and improved learning impossible. For example, in early studies of filoviruses, thousands of specimens were tested serologically for evidence of infection (*9*), with no positive results (*10*). However, new techniques would likely recover viral genetic material from those same samples (*11*), which could save time and expense invested in de novo sampling. Second, much remains to be learned from relationships between host population genetic structure and pathogen distributions. For example, some of the complexity of the distribution of Lassa fever depends on the particular lineage of *Mastomys* rodents present (*12*). Many host and vector groups currently considered single species are, in reality, complexes of species with potential (and possibly variable) epidemiologic importance. Such complexities can be explored only with detailed documentary information regarding which hosts did and did not harbor the pathogen.

Finally, and perhaps most urgent, treating biodiversity samples as disposable ignores opportunities to assemble archives of diagnostic samples for future studies. Host samples accumulated for 1 purpose could be recycled to form a strong basis for future studies of pathogens not yet known. Consider, for example, that those same samples of mammals from Africa from the early filovirus studies could have enabled quick and detailed study of *Henipavirus* distributions, in contrast to the time and effort it took to assemble other samples (*13*). Similarly, mammal samples assembled for early virus studies in West Africa (*14*) could have made possible rapid testing and evaluation of hosts for subsequent virus emergences in the region. In this sense, every biodiverse element collected as part of disease studies should be considered as potential key infrastructure for future studies, if properly documented and preserved (*15*).

Of course, biologic material that is potentially infected with dangerous pathogens carries with it some degree of responsibility, to ensure that unfortunate accidents do not occur. Two general paths are possible: 1) treatment of voucher specimen material to inactivate pathogens, such as preservation in formalin; or 2) notification by disease specialists to biodiversity specialists of any detections of pathogen-positive samples, such as samples that are inactivated or isolated. These steps are crucial, but the first option offers a way to avoid problems immediately with little extra effort.

My suggestion is not an empty dream but rather an open door. The biodiversity science community is fully prepared and willing to partner with the disease community in this effort. On the most proximate level, biodiversity specialists are eager to build scientific reference collections and are willing to curate and catalog voucher specimens. Vouchers provide permanent specimen identifiers that can be reported in publications and used to reference the voucher in genomic data bases. Furthermore, biodiversity specialists are interested in many of the same geographic regions as disease specialists and would welcome opportunities to obtain new specimen material from these regions. Finally, the role of pathogens in constraining host evolution, distribution, and ecology is of increasing interest in the biodiversity community (*16**,**17*). Many biodiversity researchers are extremely eager to explore new knowledge realms with disease specialists.
